# Porphyrin Polymers Bearing *N*,*N*′-Ethylene Crosslinkers as Photosensitizers against Bacteria

**DOI:** 10.3390/polym14224936

**Published:** 2022-11-15

**Authors:** Sofía C. Santamarina, Daniel A. Heredia, Andrés M. Durantini, Edgardo N. Durantini

**Affiliations:** IDAS-CONICET, Departamento de Química, Facultad de Ciencias Exactas, Físico-Químicas y Naturales, Universidad Nacional de Río Cuarto, Ruta Nacional 36 Km 601, Río Cuarto X5804BYA, Argentina

**Keywords:** porphyrin, polymer, photodynamic inactivation, antimicrobial, singlet oxygen

## Abstract

The appearance of microbes resistant to antibiotics requires the development of alternative therapies for the treatment of infectious diseases. In this work two polymers, **PTPPF_16_-EDA** and **PZnTPPF_16_-EDA,** were synthesized by the nucleophilic aromatic substitution of 5,10,15,20-tetrakis(pentafluorophenyl)porphyrin and its Zn(II) complex with ethylenediamine, respectively. In these structures, the tetrapyrrolic macrocycles were *N*,*N*′-ethylene crosslinked, which gives them greater mobility. The absorption spectra of the polymers showed a bathochromic shift of the Soret band of ~10 nm with respect to the monomers. This effect was also found in the red fluorescence emission peaks. Furthermore, both polymeric materials produced singlet molecular oxygen with high quantum yields. In addition, they were capable of generating superoxide anion radicals. Photodynamic inactivation sensitized by these polymers was tested in *Staphylococcus aureus* and *Escherichia coli* bacteria. A decrease in cell viability greater than 7 log (99.9999%) was observed in *S. aureus* incubated with 0.5 μM photosensitizer upon 30 min of irradiation. Under these conditions, a low inactivation of *E. coli* (0.5 log) was found. However, when the cells were treated with KI, the elimination of the Gram-negative bacteria was achieved. Therefore, these polymeric structures are interesting antimicrobial photosensitizing materials for the inactivation of pathogens.

## 1. Introduction

Since the 1950s, antibiotics have saved millions of lives both in immunocompetent and immunocompromised patients. They allowed the development of complex medical interventions and specialties that were not possible before [1]. However, the emergence of bacteria resistant to these drugs has proven to be one of the most serious concerns in recent times [2]. Antimicrobial resistance (AMR) is an ecosystem problem that raises interrelated health concerns affecting humans, animals, and the environment, as noted under the *One Health* framework [3]. This inability or reduced ability of an antimicrobial agent to inhibit the growth of a bacterium can lead to the failure of therapy for the treatment of pathogens [4]. Two phenomena were identified as the main driving forces behind the clinical problem of antibacterial resistance in human medicine. On the one hand, the imprudent use of these drugs and the inadequate administration of doses and duration of treatments facilitate the development of resistance in bacteria [5,6,7]. At the same time, pharmaceutical companies are moving away from licensing new antimicrobial drugs due to difficulties in drug development, lack of return on financial investments, and the inevitable emergence of resistant strains [8]. Therefore, antibiotic resistance found in microorganisms presents a great risk for medical practice in the whole world [9,10].

The AMR catastrophe is a prevalent multifaceted crisis that presents an appreciable challenge to the successful eradication of destructive pathogens, especially methicillin-resistant *Staphylococcus aureus* (MRSA) [11]. This microorganism is involved in widespread disease, and its multidrug resistance makes it a long-lived supergerm that can lead to devastating infections [12]. Furthermore, resistance to antimicrobials in *Escherichia coli* has caused concern for the treatment of diseases in both humans and animals, and it is considered a real problem for public health on a global scale. [3,13,14]. Therefore, *E. coli* is a significant reservoir of resistance genes that may be responsible for treatment failures in both human and veterinary medicine [15]. In this way, *E. coli* is an example of a multidrug-resistant bacterium that can be the source of extremely severe infections [16,17,18].

Therefore, the development of adequate procedures for the treatment of multi-resistant bacteria is necessary [19]. As an alternative, photodynamic inactivation (PDI) of microorganisms has been proposed as useful therapy [20]. This approach uses a photosensitizer (PS), light, and oxygen to produce highly reactive oxygen species (ROS), which can react with several cell components [21]. These molecular modifications induce a loss of biological functionality that causes cell death. Porphyrin-based PSs, both of natural and synthetic origin, have been used in the development of PDI [22,23]. However, these PSs tend to aggregate depending on the substituent groups on the tetrapyrrolic macrocycle. The formation of aggregates produces changes in the spectroscopic properties and a decrease in photodynamic activity. A possible solution to overcome these obstacles is the synthesis of PSs by forming polymers using porphyrin units as building blocks [24,25]. Consequently, to promote the efficiency of conventional porphyrins, various functionalization and modification strategies have been developed from polymers to design multifunctional photosensitizing materials [26,27]. Many porphyrin-derived PSs form polymers with one, two or three-dimensional structures. In addition, some polymers can present microporous or nanoporous assemblies formed by conjugates based on porphyrin components [28]. Therefore, porphyrin-based building block materials can combine the photochemical and photophysical properties of PS units with a versatile polymer-based structural design, which is beneficial to the PDT application [29].

Here, we report the synthesis of two polymers **PTPPF_16_-EDA** and **PZnTPPF_16_-EDA**, using as building blocks 5,10,15,20-tetrakis(pentafluorophenyl)porphyrin (**TPPF_20_**) and its Zn(II) complex (**ZnTPPF_20_**), and ethylenediamine (EDA) as crosslinker. Perfluorophenylporphyrins can be used as platforms to obtain a wide range of porphyrinoid derivatives. Furthermore, these fluorinated compounds show a high photodynamic activity with biomedical applications. The presence of fluorine atoms also improves the stability of these molecules against oxidative damage. In addition, they can be used as ^19^F magnetic resonance imaging agents for in vivo imaging in combination with fluorescence spectroscopy [30]. In the present case, the polymerization is favored because the pentafluorophenyl group substituents readily undergo a regiospecific nucleophilic aromatic substitution (S_N_Ar) reaction of the *para*-fluorine atom by a diverse set of nucleophiles [31,32,33,34]. In this case, EDA was used as the nucleophile to produce polymeric structures bearing porphyrin units. The new insight presented by this study involves polymeric photosensitizing materials composed of porphyrin units linked by a flexible spacer that improves interaction with bacterial cells. The polymeric materials were characterized by fourier-transform infrared spectroscopy (FTIR), dynamic light scattering (DLS), and scanning electron microscopy (SEM). The spectroscopic and photodynamic properties of these polymeric materials were studied and compared with their constitutive monomers in solution. Moreover, the photosensitizing ability of these compounds was evaluated for inactivation of MRSA and *E. coli* cells. These polymers are interesting materials with potential applications to eliminate bacterial pathogens.

## 2. Materials and Methods

Materials and instrumentation are described in Supplementary Materials.

### 2.1. Synthesis of PSs

**TPPF_20_** and **ZnTPPF_20_** were obtained as previously reported with some modifications [32,33].

**TPPF_20._** Pentafluorobenzaldehyde (177 mg, 0.90 mmol) and pyrrole (65 μL, 0.94 mmol) were dissolved in dichloromethane (DCM, 50 mL). The resulting solution was purged with argon for 15 min and then BF_3_·OEt_2_ (14 μL, 0.11 mmol) was added. The reaction mixture was stirred for 40 h at room temperature. After that, 2,2-dichloro-5,5-dicyano-1,4-benzoquinone (DDQ, 163 mg, 0.72 mmol) was added and the solution was stirred for 2 h at room temperature. The organic solvent was removed under reduced pressure and the crude solid was purified by flash column chromatography (silica gel, hexanes/DCM 4:1) affording 77 mg (35%) of **TPPF_20_**. TLC (hexane/DCM 4:1) R_f_ = 0.43. ^1^HNMR (CDCl_3_, TMS) d [ppm] −2.90 (s, 2H, pyrrole N-H); 8.92 (s, 8H, pyrrole-H) (Figure S1). ESI-MS [m/z] 975.0660 (975.0664 calculated for [M + H]^+^, M = C_44_H_10_F_20_N_4_). ε^Soret^ (*N*,*N*-dimethylformamide, DMF) 2.47 × 10^5^ L mol^−1^ cm^−1^.

**ZnTPPF_20._** A saturated solution of Zn(II) acetate in methanol (2 mL) was added to a solution of **TPPF_20_** (50 mg, 0.051 mmol) in DCM (10 mL). The resulting suspension was stirred for 4 h at room temperature. The formation of the metal complex was monitored by UV-visible absorption. The organic phase was washed three times with 15 mL of water. The organic solvent was evaporated under reduced pressure yielding 50 mg (95%) of **ZnTPPF_20_**. TLC (hexane/DCM 3:2) R_f_ = 0.65. ^1^HNMR (CDCl_3_, TMS) d [ppm] 8.80 (s, 8H, pyrrole-H) (Figure S1). ESI-MS [m/z] 1035.9728 (1035.9721 calculated for [M + H]^+^, M = C_44_H_8_F_20_N_4_Zn). ε^Soret^ (DMF) 2.35 × 10^5^ L mol^−1^ cm^−1^.

**PTPPF_16_-EDA**. A solution of **TPPF_20_** (17 mg, 0.017 mmol) in DMF (3 mL) was treated with EDA (24 μL, 22 mg, 0.35 mmol). The resulting mixture was sonicated until dissolution was achieved. The solution was kept in the dark without stirring at room temperature for 72 h. After that, the mixture was heated to 80 °C for 4 h. The formation of the product was examined by TLC (silica gel, hexane/DCM 4:1). The **TPPF_20_** spot disappeared while the product spot was retained at the origin. The polymer was separated by centrifugation (15 min, 3000 rpm) and the supernatant was removed. The solid was washed three times (10 mL each) with hexane and re-centrifuged. The precipitate obtained was dried under vacuum to obtain 24 mg of **PTPPF_16_-EDA**.

**PZnTPPF_16_-EDA.** This polymer was prepared using the methodology described above for **PTPPF_16_-EDA** from **Zn TPPF_20_** (36 mg, 0.035 mmol) and EDA (48 μL, 44 mg, 0.70 mmol) to yield 52 mg of **PZnTPPF_16_-EDA**. TLC (silica gel, hexane/DCM 3:2) analysis showed that the **Zn TPPF_20_** spot disappeared and the product spot was completely retained.

### 2.2. Spectroscopic Determinations

UV-visible absorption and fluorescence emission measurements in DMF were achieved as previously described [32]. Fluorescence emission spectra were recorded by exciting the samples at λ_exc_ = 424 nm. The absorbances of the compounds in DMF were 0.05 at the excitation wavelength. Emission spectra were integrated in the range between 550 and 800 nm. The fluorescence quantum yield (Φ_F_) of the PSs was determined by comparing the area under the emission spectrum, using Zn(II) 5,10,15,20-tetrakis(4-methoxyphenyl)porphyrin (**ZnTMP**) as a reference (Φ_F_ = 0.049) in DMF [35].

### 2.3. Photooxidation of 9,10-Dimethylanthracene (DMA)

Solutions containing DMA (35 µM) and PS (A = 0.1 at 424 nm) in 2 mL of DMF were irradiated with light at 424 nm (0.34 mW/cm^2^). The kinetics of DMA photooxidation were examined by analyzing the decrease in absorbance at 379 nm [32]. The observed rate constant of DMA decomposition (*k*_obs_^DMA^) for each PS was calculated from the pseudo-first order kinetic plot of ln(A_0_/A) vs. irradiation time. The quantum yield of O_2_(^1^Δ_g_) generation (Φ_Δ_) was determined by comparing the *k*_obs_^DMA^ for each PS with that for **ZnTMP** under the same experimental conditions and using **ZnTMP** as a reference (Φ_Δ_ = 0.73) [35]. Similarly, the decomposition of DMA sensitized by **TPPF_20_** and **ZnTPPF_20_** was studied, irradiating the samples with light at 414 nm (0.38 mW/cm^2^) and using **TPPF_20_** as a reference (Φ_Δ_ = 0.80) [36].

### 2.4. Photoreduction of Nitrotetrazolium Blue (NBT)

Solutions of NBT (0.2 mM), nicotinamide adenine dinucleotide (NADH, 0.5 mM) and PS (A = 0.2 at 421 nm) in 2 mL of DMF/water (5% *v*/*v*) were irradiated with white light (44 mW/cm^2^) under aerobic conditions [37]. The reduction of NBT was analyzed by the increase in absorbance at 560 nm, which is due to the appearance of the diformazan product. Control tests were performed by irradiating a porphyrin-free solution that contains NBT/NADH.

### 2.5. Bacterial Strains and Growth Conditions

Stock cultures of the MRSA (ATCC 43300) and *E. coli* (ATCC 25922) were kept in glycerol 10% (*v*/*v*) and tryptic soy (TS) broth 90% (*v*/*v*) at −80 °C [32]. Both bacterial strains were grown in TS broth at 37 °C overnight. Then, an aliquot (400 μL) of the bacterial culture was aseptically transferred to 4 mL of fresh TS broth. Cells were incubated at 37 °C until reaching the exponential phase of growth (A = 0.4 at 660 nm for *S. aureus* and A = 0.6 at 660 nm for *E. coli*). Bacteria were centrifugated (3000 rpm, 15 min) and re-suspended in an equal amount of 10 mM phosphate-buffered saline (PBS, pH = 7.4), corresponding to ~10^8^ colony forming units (CFU)/mL. After that, cells were diluted 1/10 in PBS to obtain ~10^7^ CFU/mL. Viable bacteria were counted by the spread plate technique by means of serial dilutions 10-fold in PBS. Each culture was streaked on TS agar plates in triplicate. The formation of colonies was counted after incubation of the plates for 24 at 37 °C in the dark.

### 2.6. Photosensitized Inactivation of Bacterial Suspensions

Microbial cell suspensions (2 mL, ~10^7^ CFU/mL) in PBS were treated with PS (0.5 μM) in Pyrex culture tubes (13 × 100 mm) for 30 min at 37 °C in the dark [32]. PSs were added from stock solutions (0.5 mM) in DMF. Then, 200 µL of each cell suspension was transferred to 96-well microtiter plates, which were exposed to white light (90 mW/cm^2^) for 15 and 30 min. A similar method was used to determine the PDI of *E. coli* in the presence of 100 mM KI [38]. This salt was added from 1.0 M aqueous stock solution. Bacterial cells were firstly treated with KI for 15 min at 37 °C in the dark. Then, cultures were incubated with each PS for 30 min at 37 °C in the dark. The number of viable bacterial cells was determined as previously stated above. The minimum bactericidal concentration (MBC) induced by the polymers was determined as the lowest concentration that did not show bacterial growth on the agar plate after 15 min of irradiation with white light (90 mW/cm^2^).

### 2.7. Formation of Triiodine (I_3_^−^)

Solutions containing the PS and 100 mM KI in 2 mL of DMF/10% water were irradiated with white light (44 mW/cm^2^) [38]. KI was added from a 1.0 M stock solution in water. The formation of I_3_^−^ was studied by UV-visible absorption spectroscopy through the change in absorbance at 360 nm after different irradiation times (Δt = 10 min) [39]. A Lugol’s solution was used as a positive control.

### 2.8. Controls and Statistical Analysis

*S. aureus* and *E. coli* controls were achieved using irradiated cells with white light without the PS and in the presence of PS keeping the cultures in the dark. Three separate experiments were performed to obtain the reported values. The error bars in plots represent the standard deviation. Statistically significant values were attained by one-way ANOVA at 95% confidence level (*p* < 0.05) [32].

## 3. Results and Discussion

### 3.1. Synthesis of TPPF_20_ and ZnTPPF_20_

**Scheme 1:**
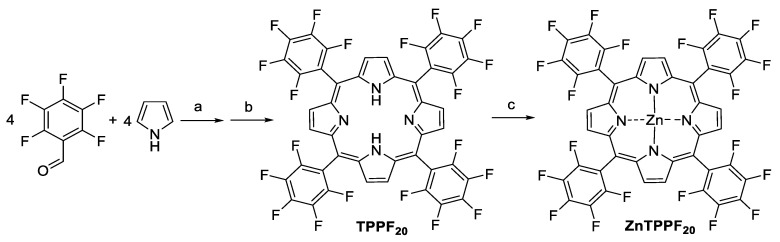
Synthesis of **TPPF_20_** and **ZnTPPF_20_**. Reagents and conditions: (**a**) BF_3_·Et_2_O, DCM, r.t., 40 h; (**b**) DDQ, DCM, r.t., 2 h, 35%; (**c**) Zn(CH_3_COO)_2_, DCM/methanol, 4 h, 95%.

The synthetic pathways to prepare the porphyrins **TPPF_20_** and **ZnTPPF_20_** are shown in Scheme 1. First, pentafluorobenzaldehyde and pyrrole were subjected to a condensation catalyzed by BF_3_·OEt_2_ in DCM for 40 h at room temperature. The hydrogenated macrocycle was oxidized with DDQ for 2 h at room temperature. The product was purified by flash column chromatography to obtain **TPPF_20_** as a purple solid in 35% yield. Similar results were reported for the synthesis of this porphyrin under similar conditions [32,40,41]. **TPPF_20_** was metaled with Zn(II) acetate in DCM/methanol to produce the metal complex **ZnTPPF_20_** in 95% yield. This reaction was carried out at room temperature for 4 h. These two synthetic steps provide in good yields the photoactive monomers with four pentafluorophenyl moieties around the macrocycle. These fluorinated porphyrins represent suitable and versatile building block units for the construction of polymeric photodynamic materials [30]. These compounds can be modified by attachment of additional substituents using the highly regioselective S_N_Ar reaction. This procedure occurs with the displacement of the four *para*-fluoro atoms in high yields [42,43].

### 3.2. Synthesis of PTPPF_16_-EDA and PZnTPPF_16_-EDA

**Scheme 2:**
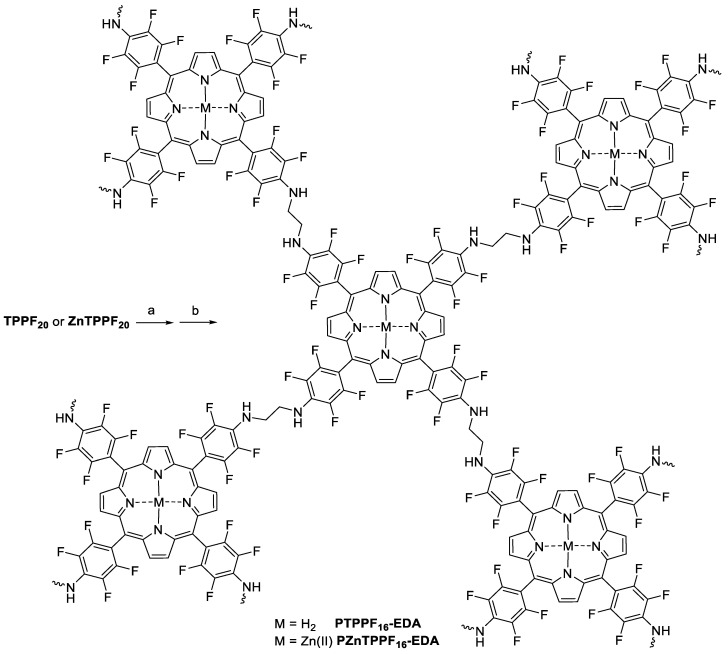
Synthesis of **PTPPF_16_-EDA** and **PZnTPPF_16_-EDA**. Reagents and conditions: (**a**) EDA, dimethylformamide, r.t., 72 h, (**b**) 80 °C 4 h.

To prepare polymeric materials based in porphyrin units, **TPPF_20_** and **ZnTPPF_20_** were reacted with EDA by S_N_Ar reaction to yield **PTPPF_16_-EDA** and **PZnTPPF_16_-EDA**, respectively. Scheme 2 shows the synthetic procedure to obtain these polymeric compounds. The reactions were carried out in DMF at room temperature for 72 h, followed by heating at 80 °C for 4 h. TLC analysis of the reaction mixtures indicates that no traces of monomeric porphyrins remain in the solutions. A new spot appears due to the formation of the polymer that is retained in the seeding site of the TLC plate. Thus, this approach produces the polymers in 100% conversion. In order to purify de polymers, they were washed several times with hexane followed by subsequent centrifugations to obtain the products **PTPPF_16_-EDA** and **PZnTPPF_16_-EDA** in high yields. Thus, the tetrapyrrolic macrocycles were *N*,*N*′-ethylene crosslinked to form the polymeric materials. This aliphatic spacer gives the porphyrin units greater mobility compared to direct covalent bonds between tetrapyrrole macrocycles, while maintaining a relatively compact polymer relative to the use of longer alkyl chains [44]. Furthermore, basic amine groups that may remain free in polymers can acquire positive charges in aqueous media [45]. These properties allow a better interaction with microorganisms, increasing the photocytotoxic action in the cells [32,46].

**Figure 1:**
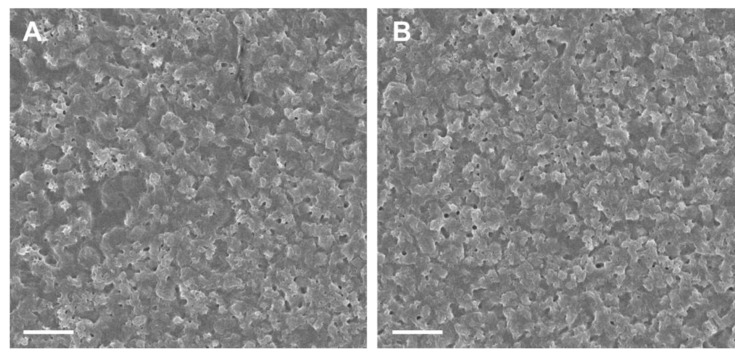
SEM images of (**A**) **PTPPF_16_-EDA** and (**B**) **PZnTPPF_16_-EDA** polymeric materials deposited as a film, scale bar 20 µm.

FT-IR spectra of **PTPPF_16_-EDA** and **PZnTPPF_16_-EDA** showed the presence of amine groups, with characteristic bands between 3600 and 3100 cm^−1^ (Figure S2) [47]. These bands are assigned to the N-H stretching vibrations of aliphatic primary amine groups and aromatic secondary amine groups. The typical bands of sp^2^ and sp^3^ C-H stretching vibrations were observed between 2980 and 2810 cm^−1^. In addition, bands of the N-H bending vibrations were found at approximately 1640 cm^−1^. DLS technology was used to measure the hydrodynamic size of the polymers in water (Figure S3). The results showed that the average sizes of **PTPPF_16_-EDA** and **PZnTPPF_16_-EDA** were 272 nm (polydispersity index = 0.30) and 347 nm (polydispersity index = 0.38), respectively. Furthermore, the conjugated polymers **PTPPF_16_-EDA** and **PZnTPPF_16_-EDA** were examined by SEM images with the aim of evaluating the shape, distribution and porosity of the polymers formed. Figure 1 exhibits representative SEM images of these polymeric materials. These images display a structure of the polymers with superimposed scales that completely cover the surface. The polymeric material shows an irregular appearance with numerous micropores on the surface. The porous material gives a larger contact surface. Comparable structures were previously found with materials formed from polymers of porphyrins [24,44,48].

### 3.3. UV-Visible Spectroscopic Characterization

**Figure 2:**
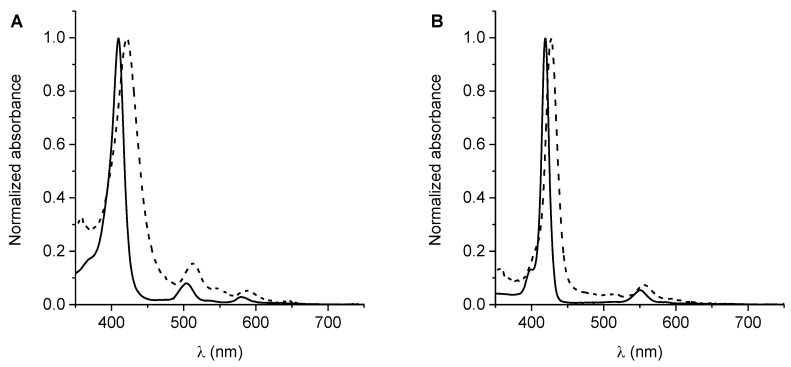
UV-visible absorption of (**A**) **TPPF_20_** (solid line) and **PTPPF_16_-EDA** (dashed line) and (**B**) **ZnTPPF_20_** (solid line) and **PZnTPPF_16_-EDA** (dashed line) in DMF.

The UV-visible absorption spectra of **TPPF_20_**, **ZnTPPF_20_**, **PTPPF_16_-EDA** and **PZnTPPF_16_-EDA** were performed in DMF (Figure 2). These spectra were also compared with that for **ZnTMP**, which was used as a reference [35]. The main optical characteristics of these compounds are summarized in Table 1. The spectrum of the free-base porphyrin **TPPF_20_** shows a *Soret* band at 410 nm and four Q bands between 590 and 650 nm. These electronic transitions are characteristics of porphyrins substituted in the *meso*-positions [49,50]. On the other hand, the UV-visible absorption spectrum of the metallated porphyrin **ZnTPPF_20_** shows a *Soret* band at 419 nm and two Q bands between 530 and 600 nm, typical of the corresponding Zn(II) substituted porphyrins [32,51]. The sharp absorption of *Soret* bands indicated that these porphyrins are dissolved as monomer in this organic solvent. Furthermore, both polymers **PTPPF_16_-EDA** and **PZnTPPF_16_-EDA** retained the spectroscopic characteristics of the porphyrin-based chromophores. The UV-visible absorption spectra confirm the polymerization of **TPPF_20_** and **ZnTPPF_20_**. The *Soret* and Q bands of both polymers exhibit a red-shifted maximum of around 10 nm in comparison with those of monomers in DMF, together with a small broadening of both bands. These results indicate only slight interaction among the porphyrin units embedded in the polymeric matrix [44]. In addition, the similarity observed between the absorption spectra of the compounds in monomeric and polymeric forms, in terms of the presence of characteristic bands, shows that these polymerized tetrapyrrolic macrocycles have electronic transitions similar to the structures in solution.

**Figure 3:**
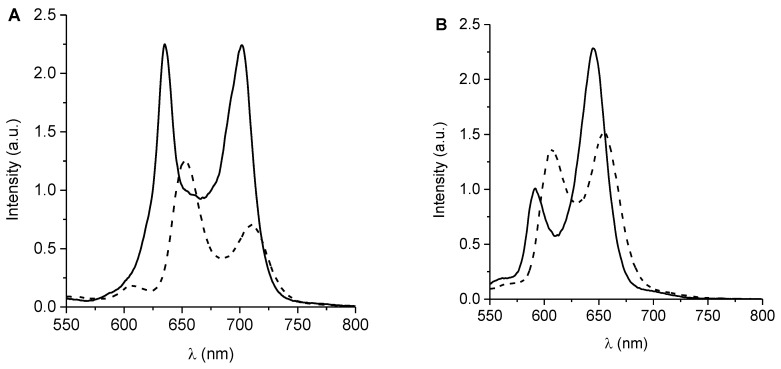
Fluorescence emission spectra of (**A**) **TPPF_20_** (solid line) and **PTPPF_16_-EDA** (dashed line) and (**B**) **ZnTPPF_20_** (solid line) and **PZnTPPF_16_-EDA** (dashed line) in DMF (λ_exc_ = 424 nm).

The fluorescence emission spectra of these PSs were measured in DMF. As can be seen in Figure 3, these compounds are capable of emitting red fluorescence. The fluorescence emission spectrum of the **TPPF_20_** shows two representative bands of porphyrins substituted at the *meso*-positions [50,52] which are located between 630 and 750 nm, while the bands of the polymer **PTPPF_20_-EDA** are bathochromically shifted by ~17 nm. Regarding **ZnTPPF_20_**, the complex exhibited two bands around 690 and 670 nm, which are distinctive for similar *meso*-substituted Zn(II) porphyrin derivatives. The corresponding polymer **PZnTPPF_16_-EDA** showed its bands with a red shift of ~15 nm. In both cases, the formation of the complex with Zn(II) produced a hypsochromic shift of ~45 nm in comparison with free-base porphyrin. These emission bands have been assigned to Q_x_(0–0) and Q_x_(0–1) transitions [32,49]. These spectroscopic properties are characteristic of porphyrins with D_2h_ symmetry, indicating that the vibronic structure of the tetrapyrrolic macrocycle remains practically unchanged upon excitation [53]. In addition, both polymers presented good emission proprieties indicating that the spectroscopic characteristics of the porphyrin-based chromophore were retained in the polymeric matrix. These results also indicate that the porphyrin can be embedded in the polymer without substantial aggregation. These minor spectral changes in absorbance spectra and the fluorescence properties of the polymers suggest that the π–π stacking between the porphyrin cores is impeded and only takes place as a weak interaction. From the absorption and fluorescence wavelength maxima of the Q_x_(0–0) bands, Stokes shifts for the polymers were determined, giving values of 6 and 10 nm for **PTPPF_16_-EDA** and **PZnTPPF_16_-EDA**, respectively. These changes indicate that small structural changes occur between the ground state and the excited singlet state of the tetrapyrrolic macrocycle due to the rigidity of the structures. Therefore, the UV-visible absorption and fluorescence emission results confirm the polymerization of porphyrins as the constitutive component of the polymer conjugates.

The values of Φ_F_ for these compounds are shown in Table 1. The Φ_F_ for **TPPF_20_** agrees with that previously reported in an organic solvent [49]. Free-base porphyrin shows a marked decrease in the intensity of its emission bands when it forms complexes with Zn(II). The Φ_F_ value decreases about 1.6 times in **ZnTPPF_20_** with respect to **TPPF_20_**. This behavior is a consequence of the presence of this metal in the tetrapyrrolic ring, which increases the formation of excited species in the triplet state [51]. The polymers presented slightly lower Φ_F_ values than their corresponding monomeric porphyrin units. Therefore, these polymeric compounds have similar properties to the corresponding porphyrins used as their building blocks.

### 3.4. Production of O_2_(^1^Δ_g_)

**Scheme 3:**
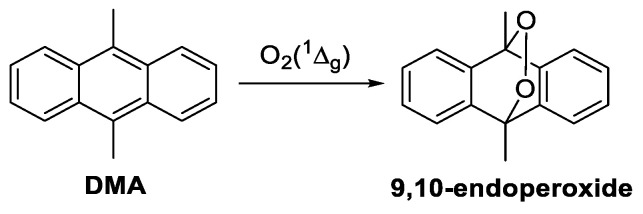
Photodecomposition of DMA mediated by O_2_(^1^Δ_g_) to produce 9,10-endoperoxide.

**Figure 4:**
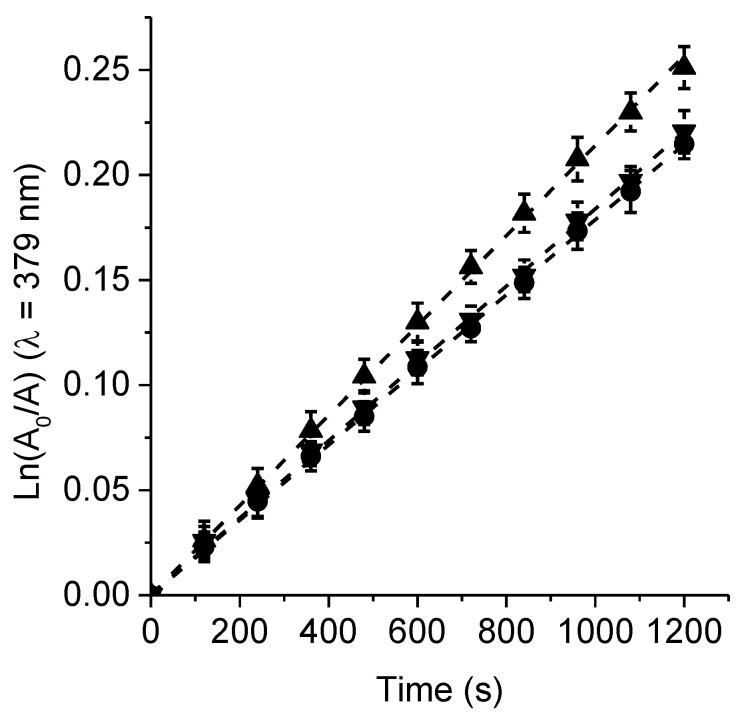
First-order plots for the photooxidation of DMA sensitized by **PTPPF_16_-EDA** (▼), **PZnTPPF_16_-EDA** (▲) and **ZnTMP** (●) in DMF, λ_irr_ = 424 nm (0.34 mW/cm^2^).

Photooxidation of DMA induced by **PTPPF_16_-EDA** and **PZnTPPF_16_-EDA** was determined in DMF (Figure 4). Samples of the anthracene derivative and each PS were irradiated at 424 nm under aerobic conditions. Furthermore, DMA decomposition sensitized by **TPPF_20_** and **ZnTPPF_20_** was investigated by irradiating the samples at 414 nm (Figure S4). The reactions were followed by the decay of the DMA band at 379 nm due to the formation of the 9,10-endoperoxide product (Scheme 3) [35,54]. The photodynamic effect induced by the polymers was compared to that of the **ZnTMP** in solution. Table 1 shows the values of *k*_obs_^DMA^ calculated from the first-order kinetic plots (Figure 4). The rate of DMA decomposition sensitized by **PTPPF_16_-EDA** was lower than that obtained for **PZnTPPF_16_-EDA**. Furthermore, the value of *k*_obs_^DMA^ obtained for **PZnTPPF_16_-EDA** increases with respect to the reference. A similar tendency was found for free-base porphyrin monomers and their complex with Zn(II). Since DMA mainly quenches O_2_(^1^Δ_g_) by chemical reaction, it was used as an approach to determine the ability of these PSs to produce O_2_(^1^Δ_g_) [54]. The Φ_Δ_ values of PSs are summarized in Figure 4. As can be seen, the formation of a chelate complex with Zn(II) produced an increase in photodynamic activity. In both cases, polymeric compounds and monomeric porphyrins, the Φ_Δ_ values were 1.15 times higher for the Zn(II) complexes than for the free-base porphyrins. This result is expected when the tetrapyrrolic macrocycle is complexed with Zn(II) [35,55]. Furthermore, Table 1 shows a slight decrease in the Φ_Δ_ values of the polymers with respect to their constitutive porphyrins. However, this reduction in O_2_(^1^Δ_g_) formation of approximately 1.06 times is largely compensated by an increase in the interaction of the polymers with the bacterial cells, which allows an increase in the photoinactivation of the microorganisms. Therefore, these polymers can be considered appropriate photosensitizing compounds with a high photodynamic capacity to produce O_2_(^1^Δ_g_) in solution.

### 3.5. Formation of O_2_^•−^

**Scheme 4:**
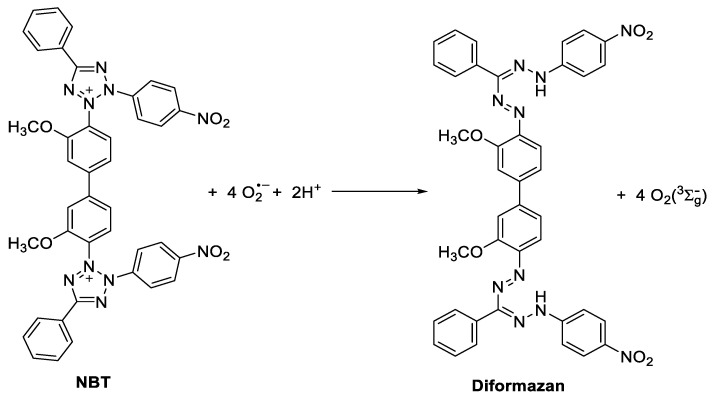
Reduction of NBT mediated by O_2_^•−^ to produce diformazan.

**Figure 5:**
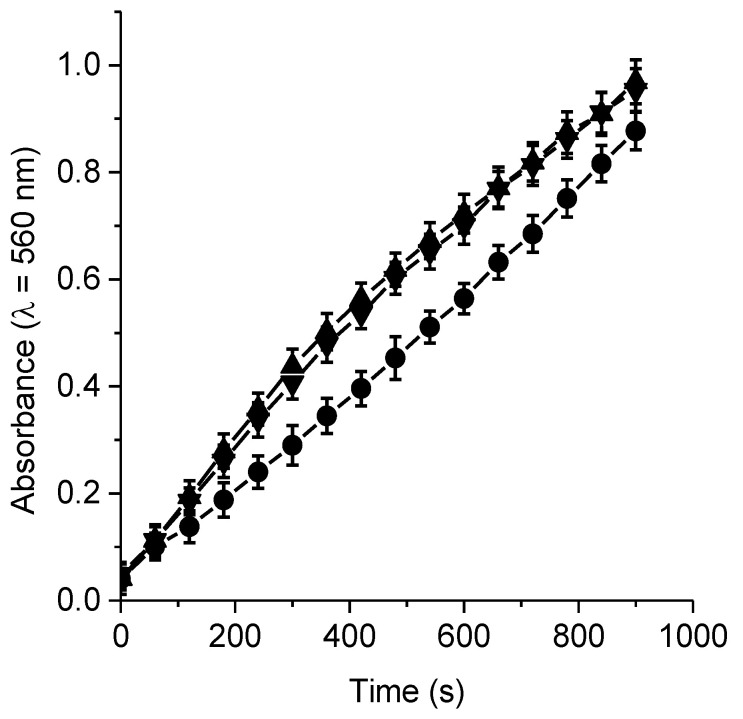
Detection of O_2_^•−^ by the NBT method as an increase in the absorption at 560 nm sensitized by **PTPPF_16_-EDA** (▼) and **PZnTPPF_16_-EDA** (▲) in DMF irradiated with white light (44 mW/cm^2^), [NBT] = 0.2 mM and [NADH] = 0.5 mM. Control of NBT + NADH without PS (●).

NBT tests were conducted to detect the presence of O_2_^•−^ in DMF/5% water. This radical reacts with NBT to produce diformazan (Scheme 4) [56], which can be detected by the absorption band at 560 nm [37,57]. Thus, solutions of **PTPPF_16_-EDA** and **PZnTPPF_16_-EDA** were irradiated with white light under aerobic conditions in the presence of NBT and the reductant NADH. As can be seen in Figure 5, the photodynamic effect sensitized by the polymers produced an increase in absorbance at 560 nm. However, the increase in absorbance was slightly greater than that produced by a solution containing NBT and NADH without the PS. Similar results were found for the reaction sensitized by **TPPF_20_** and **ZnTPPF_20_** (Figure S5). It was observed that the generation of diformazan increases by 0.1 and 0.2 absorbance units after 15 min of irradiation in the presence of the polymeric materials and the constitutional porphyrins, respectively. Although the differences in absorbance are significant, these values are below those previously reported for porphyrin derivatives and conjugate polymers [37,44,57].

Therefore, these compounds are mainly efficient sensitizers of O_2_(^1^Δ_g_) in solution. However, although to a lesser extent, these polymers are also capable of generating O_2_^•−^ especially in the presence of NADH [58]. The main pathways of photodynamic action determined in solution can change significantly in a cellular medium, depending on the polarity of the microenvironment and the availability of substrates where the PS is located. Therefore, it is difficult to make correlations between photodynamic data obtained in solution with those in microbial cells.

### 3.6. Photoinactivation of Bacterial Cell Suspensions

**Figure 6:**
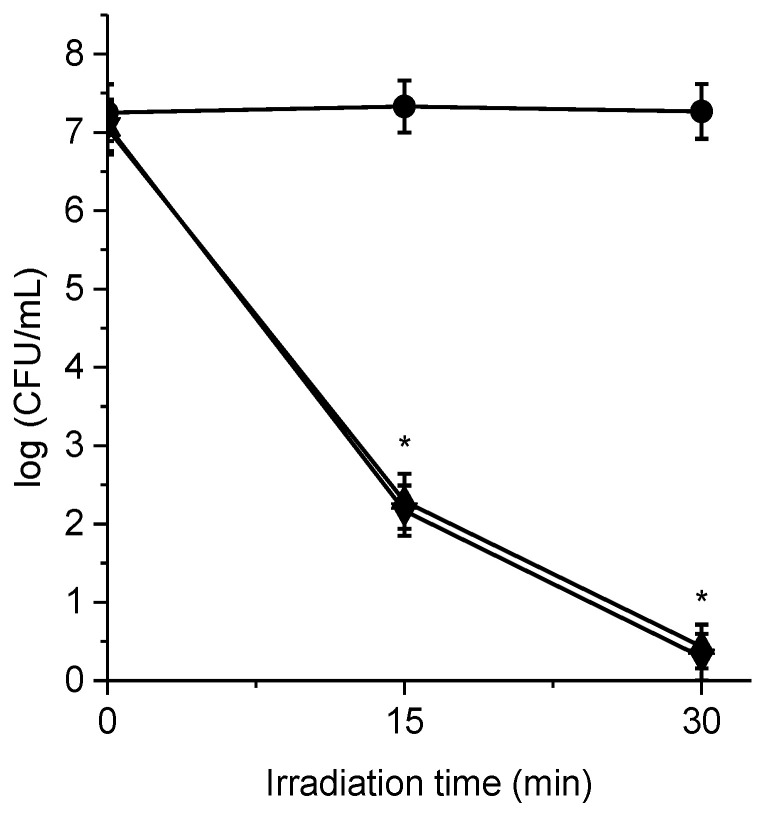
Survival of S. aureus (~10^7^ CFU/mL) treated with 0.5 µM (▼) **PTPPF_16_-EDA** and (▲) **PZnTPPF_16_-EDA** for 30 min at 37 °C in the dark and irradiated with white light (90 mW/cm^2^) for different times. Irradiated control: culture without PS (●) (* *p* < 0.05 compared with control).

**Figure 7:**
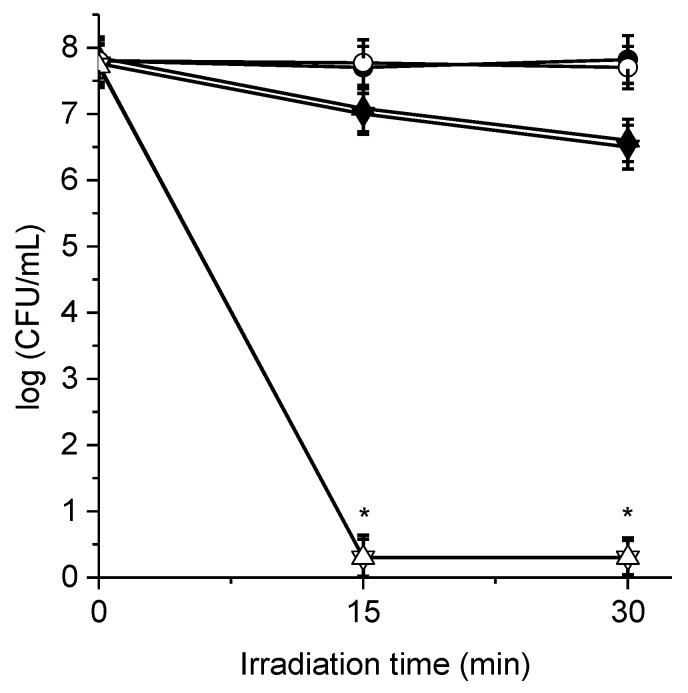
Survival of E. coli (~10^7^ CFU/mL) treated with 0.5 µM (▼) **PTPPF_16_-EDA** or (▲) **PZnTPPF_16_-EDA** for 30 min at 37 °C in the dark and irradiated with white light (90 mW/cm^2^) for different times. Cells incubated with 100 mM KI for 20 min at 37 °C in the dark prior to PDI treatments with (▽) **PTPPF_16_-EDA** or (△) **PZnTPPF_16_-EDA**. Irradiated controls: culture without PS (●) and culture treated with 100 mM KI without PS (O) (* *p* < 0.05 compared with control).

Photosensitized inactivation of *S. aureus* and *E. coli* was investigated after different irradiation periods (15 and 30 min) with white light (90 mW/cm^2^) in PBS cell suspensions. These bacteria were selected as representatives of Gram-positive and Gram-negative pathogens that cause numerous diseases in humans and animals [3,11]. Both bacterial strains were incubated with 0.5 µM PS for 30 min in the dark. The viability of the microbial cells was not affected by cells being treated with PS in the dark (Figures S6–S9). Furthermore, no toxicity was observed in irradiated cells in the absence of PS (Figure 6 and Figure 7).

In the case of *S. aureus* (Figure 6), the photodynamic activity induced by **PTPPF_16_-EDA** and **PZnTPPF_16_-EDA** in PSs produced a 5 log reduction in the cell survival upon 15 min of irradiation. Under these conditions, the MBC was determined giving a value of 0.30 ± 0.05 μM for both polymers, which represents 3.4 × 10^−4^ mg/mL and 3.6 × 10^−4^ mg/mL of **PTPPF_16_-EDA** and **PZnTPPF_16_-EDA**, respectively. After 30 min of irradiation, *S. aureus* cells were completed eradicated with a reduction of more than 7 log in cell viability. No significant difference was observed between the photoinactivation induced by the free-base polymer or its complex with Zn(II). Under these conditions, cell death was greater than 99.9999% of the bacterial population. In contrast, the constitutive monomers, **TPPF_20_** and **ZnTPPF_20_**, produced a decrease of 1.5 log after 30 min of irradiation (Figure S10). This significant difference in photoinactivation demonstrates the importance of the polymeric material in the inactivation of *S. aureus*. In previous investigations, similar results to those sensitized by **PTPPF_16_-EDA** and **PZnTPPF_16_-EDA** were found for PSs derived from porphyrins substituted by positively charged precursor groups [38,45]. Comparable photokilling results were also found for porphyrin derivatives as monomers substituted by precursor groups of positive charges, although using a higher concentration. Furthermore, the photodynamic action on *S. aureus* was comparable to that produced by a conjugated polymer based on a Zn(II) porphyrin [44].

Survival of *E. coli* cells treated with the polymeric PSs upon irradiation is shown in Figure 7. An approximately 1.3 log decrease in bacterial survival was found after 30 min of irradiation. There was also no difference between photodamage induced by **PTPPF_16_-EDA** or **PZnTPPF_16_-EDA** in *E. coli*. Under these conditions, photokilling induced by **TPPF_20_** and **ZnTPPF_20_** was negligible relative to the irradiated control (Figure S11). This low photoinactivating activity can be attributed to the fact that Gram-negative bacteria are more difficult to kill than Gram-positive ones due to the significant differences in the structure of their cell walls [21,59]. In general, in vitro studies with microorganisms indicate that Gram-positive bacteria are susceptible to the effect produced by a wide variety of PSs, including those that are neutral or anionic. In contrast, Gram-negative bacteria are resistant to a wide variety of photosensitizing compounds. Therefore, this type of microbe is the most challenging target for any type of antimicrobial treatment [20,22]. The outer membrane of Gram-negative bacteria has an effective permeability barrier between the cell and the surrounding environment, which tends to restrict the binding and penetration of many PSs [21].

Although there are a considerable number of studies of polymer-conjugated porphyrins for the photoinactivation of microorganisms, data on the applications of the porphyrin-based building block materials are quite scarce [26,27]. Porphyrin-based covalent organic frameworks by Schiff-base chemistry were obtained as photosensitizing agents [60]. The three-dimensional structures presented an effective antibacterial activity toward *Pseudomonas aeruginosa* and *Enterococcus faecalis* biofilms. Furthermore, covalent organic frameworks containing were formed from 5,10,15,20-tetrakis(4-aminophenyl)porphyrin linked by different aromatic spacers [61]. These PSs exhibited superior antibacterial effects toward *S. aureus* and *E. coli*. Moreover, poly(2-hydroxyethylmethacrylate) derivatives bearing porphyrinic units were synthesized as photoactive materials [62]; these polymeric PSs showed bactericidal properties against *S. aureus* and *E. coli*. Likewise, a conjugated polymer based on Zn(II) porphyrin was obtained by the homocoupling reaction of terminal alkyne groups [44]. This material was able to eliminate *S. aureus* cells using a low concentration and a short irradiation time. Furthermore, complete inactivation of *E. coli* was achieved when PDI was potentiated with KI.

To enhance the photoinactivation of *E. coli* sensitized by **PTPPF_16_-EDA** and **PZnTPPF_16_-EDA**, bacterial PDI was investigated in the presence of KI. Thus, cultures were incubated with 100 mM KI for 20 min in the dark before treatment with PSs. These concentrations of KI were chosen considering previous results for the potentiation of PDI [38,63]. This inorganic salt was not toxic for *E. coli* cells exposed to irradiation for 30 min (Figure 7). Moreover, survival of *E. coli* cells was not modified by cultures incubated with 100 mM KI and PS in the dark (Figure S7). The combined effect of the photodynamic action and the addition of KI produced an increase in the photoinactivation of about 7 log (Figure 7). Under these conditions, a complete elimination of the bacteria was achieved after 15 min of irradiation. A similar killing effect potentiated by iodide anions was found for both polymers. In the presence of 100 mM KI and upon an irradiation of 15 min, a MBC value of 0.40 ± 0.05 μM was found for these PSs, equivalent to 4.5 × 10^−4^ and 4.8 × 10^−4^ mg/mL for **PTPPF_16_-EDA** and **PZnTPPF_16_-EDA**, respectively. It was reported that the efficacy of PDI in *E. coli* mediated by different PSs can be significantly increased by the addition of KI [64,65,66,67]. Iodide anion enhancement was also used to improve the PDI of bacteria sensitized by various porphyrin derivatives [38,68,69,70]. In addition, this procedure was used to increase the photoinactivating activity of polymers in solution and deposited on a surface [44,71].

**Figure 8:**
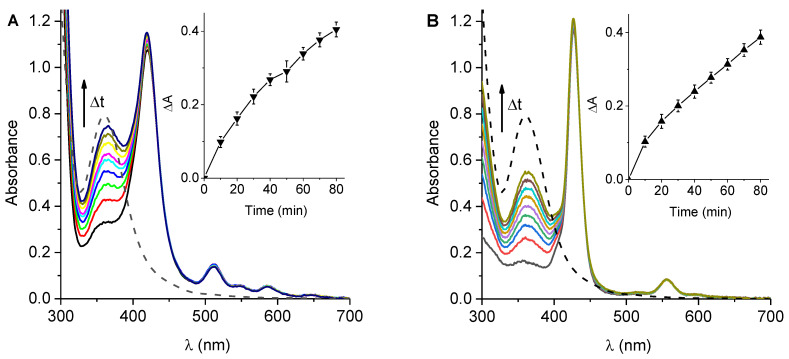
Absorption spectra of (**A**) **PTPPF_16_-EDA** and (**B**) **PZnTPPF_16_-EDA** containing 100 mM KI in DMF/10% after different irradiation times (Δt = 10 min, solid lines) with white light (44 mW/cm^2^) and Lugol’s solution (dashed line). Inset: changes in absorbance at 360 nm after different irradiation times.

The formation of iodine was determined in solutions of **PTPPF_16_-EDA** and **PZnTPPF_16_-EDA** containing 100 mM KI in DMF/10% water. Samples were irradiated with white light for different times and the generation of iodine was sensed by the UV-visible absorption spectra. As shown in Figure 8, the appearance of a new band centered at 360 nm was found upon irradiation, which increases with periods of light exposure. This band was assigned to the formation of iodine in this medium [39,72]. In addition, the spectra were compared with that of a diluted Lugol’s solution as a positive control. Figure 8 insets show the increase in the absorbance at 360 nm after different irradiation times. The absorbance at this wavelength increased gradually as the white light exposure time elapsed for the KI-containing solutions of both polymers. These results agree with the formation of O_2_(^1^Δ_g_) of these polymeric compounds. Similar behavior was also observed using solutions of porphyrins with the addition of KI [38,70]. The reaction of iodide anions and O_2_(^1^Δ_g_) produces triiodide anions (I_3_^−^) in aqueous media (Scheme S1). In addition, this process can form hydrogen peroxide (H_2_O_2_), which reacts with iodide anions to produce I_3_^−^ [73,74]. Therefore, light activation of the polymers, **PTPPF_16_-EDA** or **PZnTPPF_16_-EDA**, leads to the formation of O_2_(^1^Δ_g_). This ROS interacts with KI to produce biocides I_2_ or I_3_^−^, which enhances bacterial inactivation [75]. Consequently, this alternative pathway of cytotoxicity can be used to enhance the PDI of microbial cells. Therefore, the addition of KI allowed potentiation of the PDI sensitized by photodynamic polymers of Gram-negative bacteria.

## 4. Conclusions

Two new polymers, **PTPPF_16_-EDA** and **PZnTPPF_16_-EDA,** built from units of porphyrin were conveniently obtained. This approach involves the use of two easily synthetized porphyrins, **TPPF_20_** and its complex with Zn(II). The S_N_Ar reaction of these porphyrins with the EDA nucleophile was used to obtain the polymeric materials in high yields. Furthermore, this linker allows the union between the tetrapyrrolic macrocycles, leaving a flexible aliphatic spacer that provides greater mobility of the polymeric structures. Spectroscopic studies show a bathochromic shift of the absorption and emission bands of the polymers with respect to the monomeric constitutional units. The photodynamic activity presents an important contribution of a type II mechanism, with high production of O_2_(^1^Δ_g_). Furthermore, these polymers can produce O_2_^•−^ in the presence of NADH. On the other hand, these polymeric compounds were tested as photosensitizing agents to inactivate bacteria. Both polymers were able to eliminate *S. aureus* when cultures were incubated with 0.5 μM PS upon 15 min of irradiation. These conditions using both a low concentration of the polymer and low fluence of white light are appropriated for PDI treatments. In contrast, the photodynamic action sensitized by the polymers was poorly effective in inactivating *E. coli*. However, the addition of KI considerably improved the antimicrobial activity against Gram-negative bacteria. Thus, potentiation with KI made it possible to obtain an eradication of *E. coli* similar to that obtained for Gram-positive bacteria. This increase can be produced by the formation of reactive iodine species, mainly I_3_^−^, sensitized by the polymers under aerobic conditions. Therefore, these polymers are interesting photodynamic structures with potential applications as antimicrobial agents to kill pathogenic bacteria.

## Figures and Tables

**Table 1:** Spectroscopic and photodynamic properties of PSs in DMF.

PS	λ^Soret^ (nm)	λ_em_ (nm)	Φ_F_ ^a^	*k*_obs_^DMA^ (s^−1^)	Φ_Δ_ ^d^
**TPPF_20_**	410	635	0.039 ± 0.003	(2.23 ± 0.04) × 10^−4 b^	0.80 ± 0.03 ^e^
**ZnTPPF_20_**	419	592	0.025 ± 0.002	(2.83 ± 0.05) × 10^−4 b^	0.92 ± 0.04 ^e^
**PTPPF_16_-EDA**	421	653	0.030 ± 0.003	(1.84 ± 0.03) × 10^−4 c^	0.75 ± 0.03 ^f^
**PZnTPPF_16_-EDA**	428	607	0.018 ± 0.002	(2.14 ± 0.04) × 10^−4 c^	0.87 ± 0.04 ^f^

^a^ Fluorescence quantum yields using **ZnTMP** as the reference (Φ_F_ = 0.049) [35], ^b^ λ_irr_ = 414 nm, ^c^ λ_irr_ = 424 nm, ^d^ quantum yield of O_2_(^1^Δ_g_) production, ^e^ using **TPPF_20_** as the reference (Φ_F_ = 0.80) [36], ^f^ using **ZnTMP** as the reference *k*_obs_^DMA^ = (1.79 ± 0.02) × 10^−4^ s^−1^ (Φ_F_ = 0.73) [35].

## Data Availability

Not applicable.

## Supplementary Materials

The following supporting information can be downloaded at: https://www.mdpi.com/article/10.3390/polym14224936/s1, Materials; Instrumentation; Figure S1: ^1^HNMR spectra of **PTPPF_16_-EDA** and **PZnTPPF_16_-EDA** in CDCl_3_; Figure S2: FT-IR spectra of **PTPPF_16_-EDA** and **PZnTPPF_16_-EDA**; Figure S3: DLS profile of **PTPPF_16_-EDA** and **PZnTPPF_16_-EDA** in water; Figure S4: First-order plots for the photooxidation of DMA sensitized by **TPPF_20_** and **ZnTPPF_20_** in DMF, λ_irr_ = 414 nm (0.38 mW/cm^2^); Figure S5: Detection of O_2_^•−^ by the NBT method as an increase in the absorption at 560 nm sensitized by **TPPF_20_** and **ZnTPPF_20_** in DMF irradiated with white light (44 mW/cm^2^), [NBT] = 0.2 mM and [NADH] = 0.5 mM. Control of NBT + NADH without PS; Figure S6: Survival of *S. aureus* (~10^7^ CFU/mL) treated with 0.5 µM **PTPPF_16_-EDA** and **PZnTPPF_16_-EDA** for 30 min at 37 °C in the dark and kept in the dark for different times; Figure S7: Survival of *E. coli* (~10^7^ CFU/mL) treated with 0.5 µM **PTPPF_16_-EDA** and **PZnTPPF_16_-EDA** for 30 min at 37 °C in the dark and kept in the dark for different times. Cells incubated with 100 mM KI for 20 min at 37 °C in the dark prior to PDI treatments with **PTPPF_16_-EDA** and **PZnTPPF_16_-EDA**; Figure S8: Survival of *S. aureus* (~10^7^ CFU/mL) treated with 0.5 µM **TPPF_20_** and **ZnTPPF_20_** for 30 min at 37 °C in the dark and kept in the dark for different times; Figure S9: Survival of *E. coli* (~10^7^ CFU/mL) treated with 0.5 µM **TPPF_20_** and **ZnTPPF_20_** for 30 min at 37 °C in the dark and kept in the dark for different times; Figure S10: Survival of *S. aureus* (~10^7^ CFU/mL) treated with 0.5 µM **TPPF_20_** and **ZnTPPF_20_** for 30 min at 37 °C in the dark and irradiated with white light (90 mW/cm^2^) for different times. Irradiated control: culture without PS (* *p* < 0.05 compared with control); Figure S11: Survival of E. coli (~10^7^ CFU/mL) treated with 0.5 µM **TPPF_20_** and **ZnTPPF_20_** for 30 min at 37 °C in the dark and irradiated with white light (90 mW/cm^2^) for different times. Irradiated controls: culture without PS (* *p* < 0.05 compared with control); Scheme S1: Reaction of O_2_(^1^Δ_g_) with iodide anions in aqueous media [35,38,44,67].
