# Supplementary material for: Porphyrin Polymers Bearing N,N′-Ethylene Crosslinkers as Photosensitizers against Bacteria
Source: Polymers (Basel). 2022 Nov 15;14(22):4936. doi: 10.3390/polym14224936 (PMC9696963; doi:10.3390/polym14224936)
Supplement: Supplementary file 1 [file polymers-14-04936-s001.zip › polymers-1991938-supplementary.pdf]

Supplementary Materials

Porphyrin polymers bearing *N,N'*-ethylene crosslinkers as photosensitizers against bacteria

Sofía C. Santamarina[1], Daniel A. Heredia[1], Andrés M. Durantini[1] and Edgardo N. Durantini[1]*

[1] IDAS-CONICET, Departamento de Química, Facultad de Ciencias Exactas, Físico-Químicas y Naturales, Universidad Nacional de Río Cuarto, Ruta Nacional 36 Km 601, X5804BYA Río Cuarto, Córdoba, Argentina.

Table of Contents

1. Materials Page S2

2. Instrumentation Page S2

3. Scheme and figures Page S4

4. References Page S10

* Correspondence: edurantini@exa.unrc.edu.ar (E.N.D.)

1. Materials

Compounds were obtained from Sigma-Aldrich (Milwaukee, WI, USA) that were used without further purification. Organic solvents (GR grade) from Merck (Darmstadt, Germany) were distilled and maintained on molecular sieves. Ultrapure water was obtained from a Labconco (Kansas City, MO, USA) equipment model 90901-01. Silica gel thin-layer chromatography (TLC) plates (250 microns) were acquired from Analtech (Newark, DE, USA) and silica gel 60 (0.040-0.063 mm, 230-400 mesh) from Merck (Darmstadt, Germany). Tryptic soy (TS) broth and agar from Britania (Buenos Aires, Argentina) were used in microbial cultures. Microtiter plates (96-well) were acquired from Deltalab (Barcelona, Spain). Zn(II) 5,10,15,20-tetrakis(4-methoxyphenyl)porphyrin was synthesized as previously described [1].

2. Instrumentation

Proton nuclear magnetic resonance spectra were performed on a FT-NMR Bruker Advance DPX400 at 400 MHz (Bruker BioSpin, Rheinstetten, Germany). Mass spectra were recorded on a Bruker micrOTOF-QII (Bruker Daltonics, Billerica, MA, USA) equipped with an ESI source (ESI-MS). IR spectra were recorded on a Bruker Tensor 27 FT-IR (Ettlingen, Germany) in KBr pellets. Hydrodynamic diameters were measured by dynamic light scattering (DLS, Malvern 4700 with goniometer, Malvern, Worcestershire, United Kingdom) operating with an OBIS 488 nm solid state laser source (Coherent, Santa Clara, CA, USA). Scanning electron microscopy (SEM) images were obtained with a field emission scanning electron microscope FE-SEM (Σigma Zeiss, Oberkochen, Germany) with a thin Cr film on the sample surface and an acceleration voltage of 5 kV. Containers were printed using a Prusa i3 MK3S 3D printer purchased from Prusa Research (Praga, Czech Republic). UV-visible absorption spectra were carried out on a Shimadzu UV-2401PC spectrometer (Shimadzu Corporation, Tokyo, Japan). Fluorescence emission spectra were performed on a Spex FluoroMax spectrofluorometer (Horiba Jobin Yvon Inc, Edison, NJ, USA). Spectroscopic determinations were performed in a quartz cell of 1 cm path length at room temperature. A

Radiometer Laser Mate-Q (Coherent, Santa Clara, CA, USA) was used to determine the light fluence rates. Steady-state photolysis of 9,10-dimethylanthracene (DMA) were performed with a Cole-Parmer illuminator 41720-series (150 W halogen lamp, Cole-Parmer, Vernon Hills, IL, USA) in combination with a high intensity grating monochromator (Photon Technology Instrument, Birmingham, NJ, USA) [2]. This arrangement produces a light fluence rate of 0.34 mW/cm^2 at 424 ± 6 nm and 0.38 mW/cm^2 at 414 ± 6 nm. Nitro blue tetrazolium (NBT) method and generation of iodine species were performed irradiating the sample with white light (Cole-Parmer illuminator 41720-series, 350-800 nm) filtered through a 2.5 cm glass cuvette filled with water giving a light fluence rate of 44 mW/cm^2. Cell suspensions were irradiated with a Novamat 130 AF (Braun Photo Technik, Nürnberg, Germany) projector containing a 150 W lamp. A 2.5 cm glass cuvette filled with water without circulation was used to remove the heat from the lamp. A wavelength range between 350 and 800 nm was selected by optical filters. The projector was placed vertically with the light beam focused on the 96-well microtiter plate lid, producing a fluence rate of 90 mW/cm^2 [3].

3. Scheme and figures

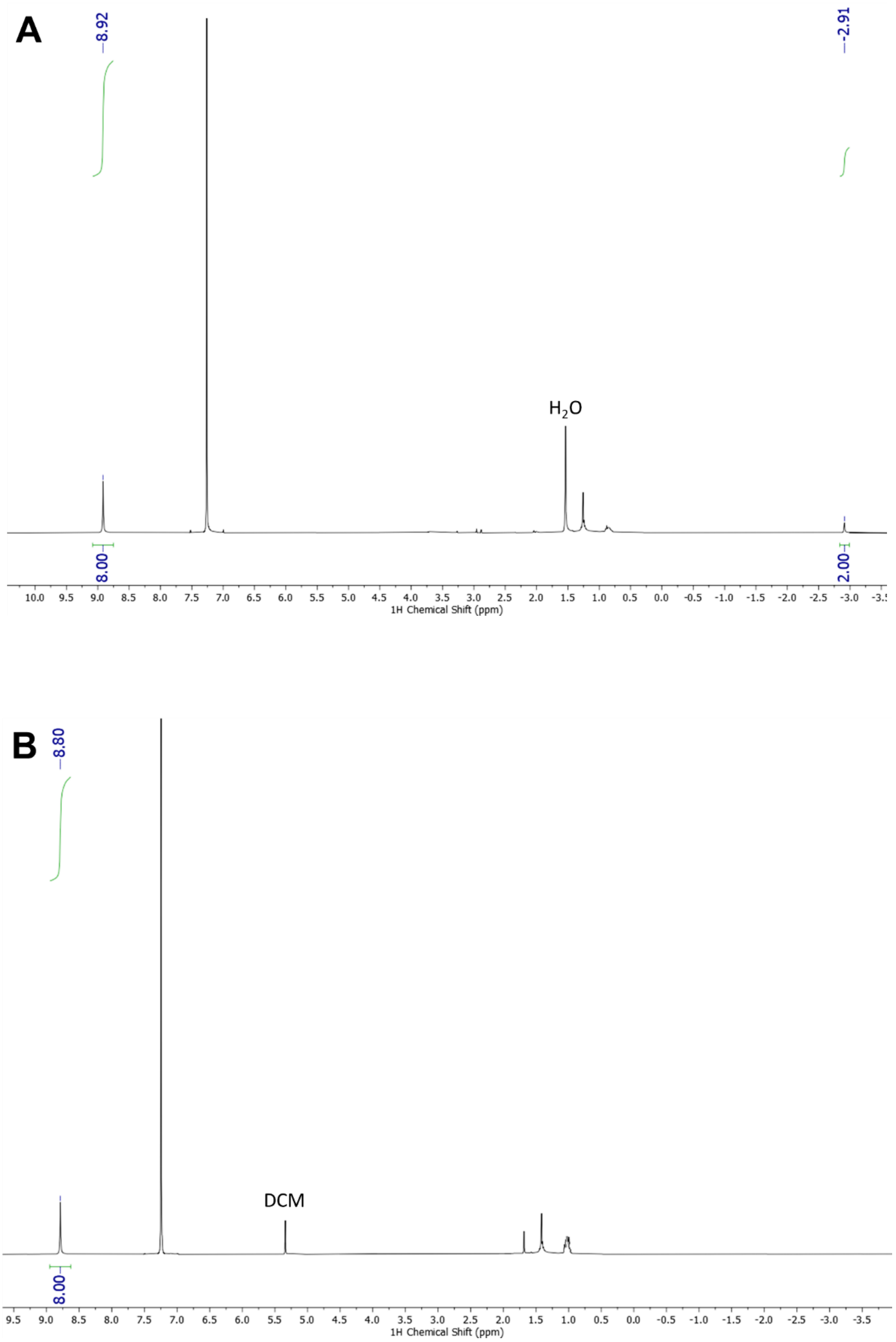

Figure S1. ^{1}HNMR spectra of (A) **PTPPF$_{16}$-EDA** and (B) **PZnTPPF$_{16}$-EDA** in $CDCl_3$**.**

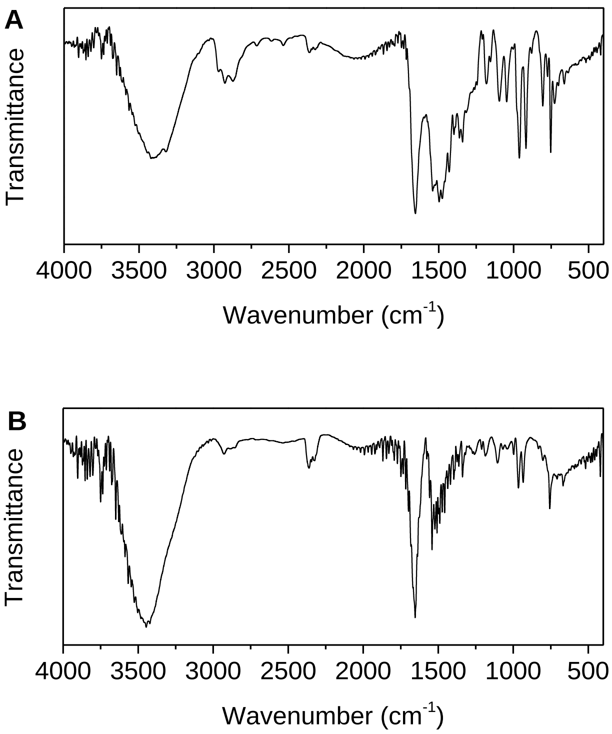

Figure S2. FT-IR spectra of (A) **PTPPF16-EDA** and (B) **PZnTPPF16-EDA**.

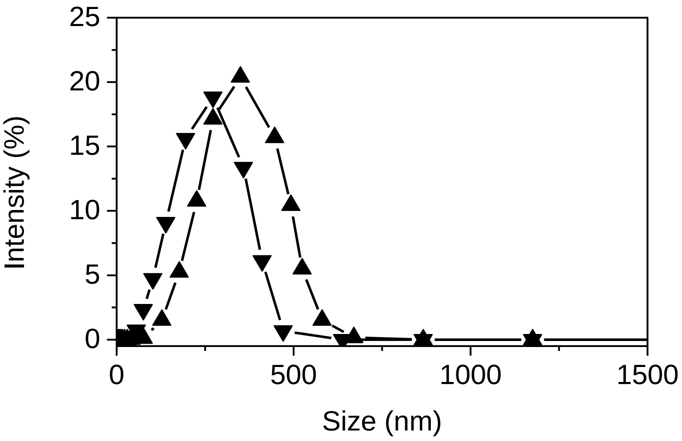

Figure S3. DLS profile of **PTPPF16-EDA** (▼) and **PZnTPPF16-EDA** (▲) in water.

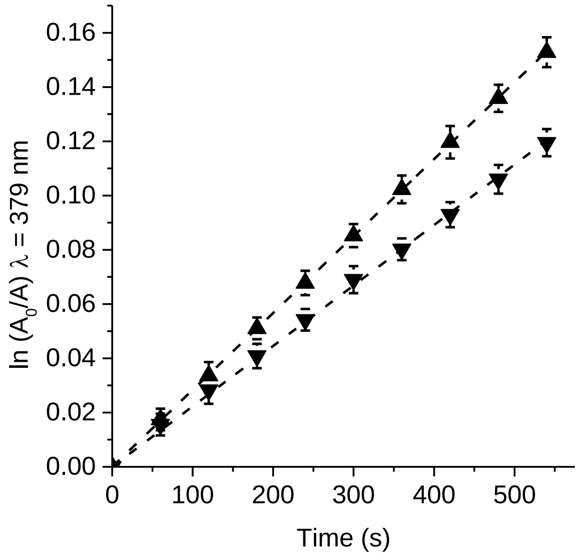

Figure S4. First-order plots for the photooxidation of DMA sensitized by **TPPF20** (▼) and **ZnTPPF20** (▲) in DMF, λ_{irr} = 414 nm (0.38 mW/cm^2).

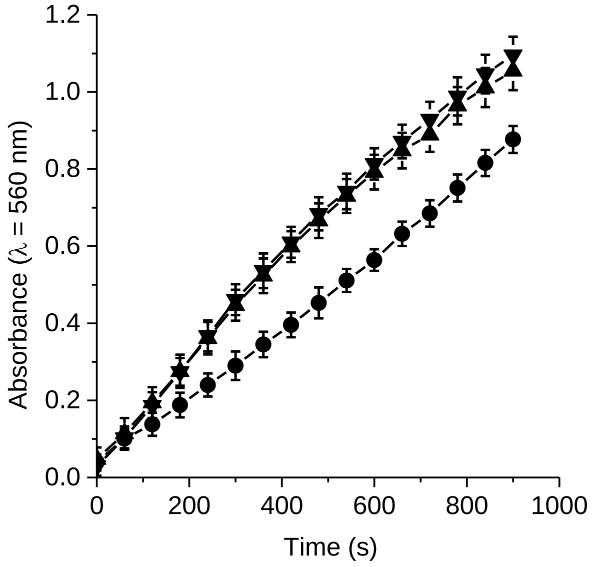

Figure S5. Detection of $O_2^{\bullet-}$ by the NBT method as an increase in the absorption at 560 nm sensitized by **TPPF20** (▼) and **ZnTPPF20** (▲) in DMF irradiated with white light (44 mW/cm^2), [NBT] = 0.2 mM and [NADH] = 0.5 mM. Control of NBT + NADH without PS (●).

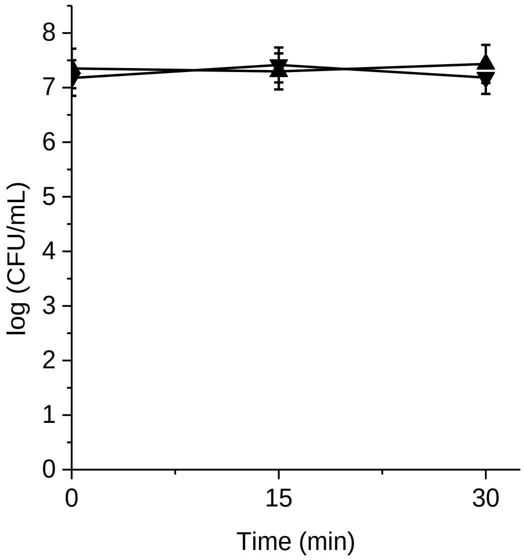

Figure S6. Survival of *S. aureus* (~10^7 CFU/mL) treated with 0.5 µM (▼) **PTPPF16-EDA** and (▲) **PZnTPPF16-EDA** for 30 min at 37 ºC in the dark and kept in the dark for different times.

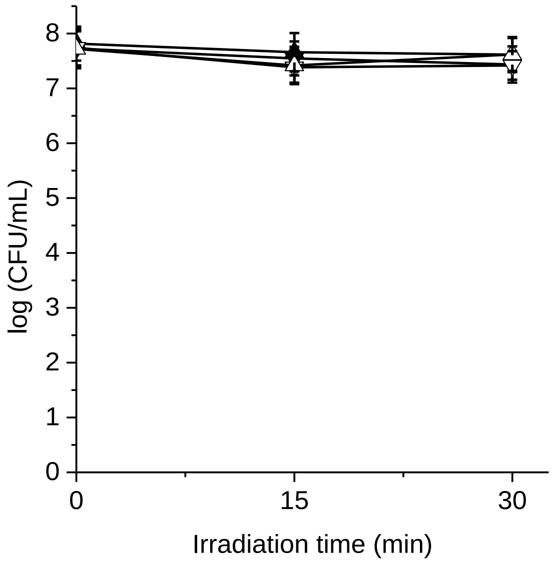

Figure S7. Survival of *E. coli* (~10^7 CFU/mL) treated with 0.5 µM (▼) **PTPPF16-EDA** and (▲) **PZnTPPF16-EDA** for 30 min at 37 ºC in the dark and kept in the dark for different times. Cells incubated with 100 mM KI for 20 min at 37 ºC in the dark prior to PDI treatments with (▽) **PTPPF16-EDA** and (△) **PZnTPPF16-EDA**.

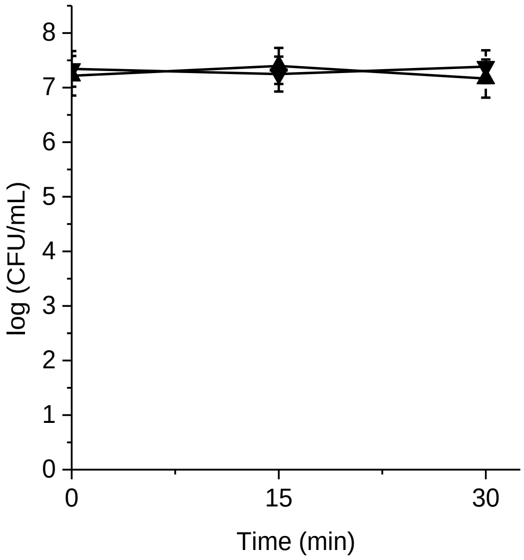

Figure S8. Survival of *S. aureus* (~10^7 CFU/mL) treated with 0.5 µM (▼) **$TPPF_{20}$** and (▲) **$ZnTPPF_{20}$** for 30 min at 37 ºC in the dark and kept in the dark for different times.

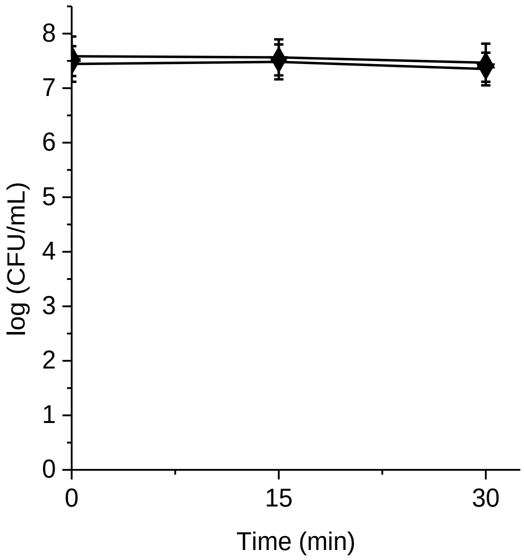

Figure S9. Survival of *E. coli* (~10^7 CFU/mL) treated with 0.5 µM (▼) **$TPPF_{20}$** and (▲) **$ZnTPPF_{20}$** for 30 min at 37 ºC in the dark and kept in the dark for different times.

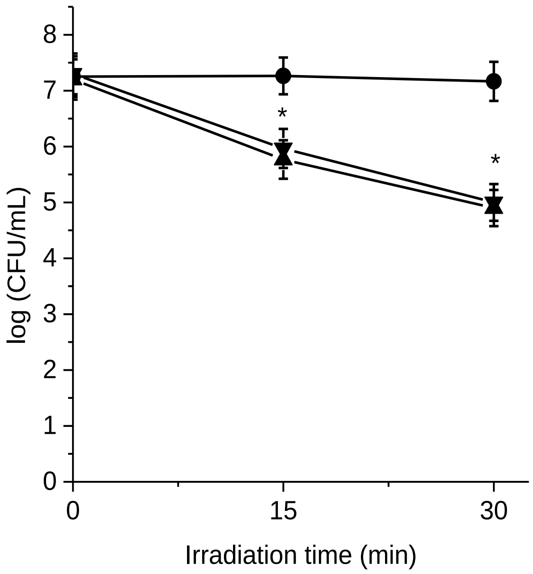

Figure S10. Survival of *S. aureus* (~10^7 CFU/mL) treated with 0.5 µM (▼) **TPPF$_{20}$** and (▲) **ZnTPPF$_{20}$** for 30 min at 37 ºC in the dark and irradiated with white light (90 mW/cm^2) for different times. Irradiated control: culture without PS (●) (*p < 0.05 compared with control).

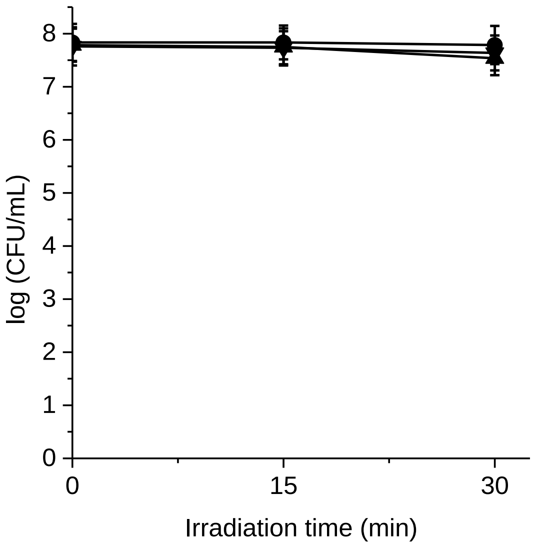

Figure S11. Survival of *E. coli* (~10^7 CFU/mL) treated with 0.5 µM (▼) **TPPF$_{20}$** and (▲) **ZnTPPF$_{20}$** for 30 min at 37 ºC in the dark and irradiated with white light (90 mW/cm^2) for different times. Irradiated controls: culture without PS (●) (*p < 0.05 compared with control).

$$O_2(^1\Delta_g) + I^- + H_2O \longrightarrow IOOH + OH^-$$

$$IOOH + I^- \longrightarrow I_2 + HO_2^-$$

$$I_2 + I^- \longrightarrow I_3^-$$

$$HO_2^- + H_2O \longrightarrow H_2O_2 + OH^-$$

$$H_2O_2 + 2I^- + 2H^+ \longrightarrow I_2 + 2H_2O$$

Scheme S1. Reaction of $O_2(^1\Delta_g)$ with iodide anions in aqueous media [4].

4. References

1. Milanesio, M. E.; Alvarez, M. G.; Yslas, E. I.; Borsarelli, C. D.; Silber, J. J.; Rivarola, V.; Durantini, E. N. Photodynamic studies of metallo 5,10,15,20-tetrakis(4-methoxyphenyl) porphyrin: photochemical characterization and biological consequences in a human carcinoma cell line. *Photochem. Photobiol.* **2001**, *74*, 14-21.

2. Pérez, M. E.; Durantini, J. E.; Reynoso, E.; Alvarez, M. G.; Milanesio, M. E.; Durantini, E. N. Porphyrin-schiff base conjugates bearing basic amino groups as antimicrobial phototherapeutic agents. *Molecules* **2021**, *26*, 5877.

3. Santamarina, S. C.; Heredia, D. A.; Durantini, A. M.; Durantini, E. N. Antimicrobial photosensitizing material based on conjugated Zn(II) porphyrins. *Antibiotics* **2022**, *11*, 91.

4. Reynoso, E.; Quiroga, E. D.; Agazzi, M. L.; Ballatore, M. B.; Bertolotti, S. G.; Durantini E. N. Photodynamic inactivation of microorganisms sensitized by cationic BODIPY derivatives potentiated by potassium iodide. *Photochem. Photobiol. Sci.* **2017**, *16*, 1524-1536.
